# Distribution of Brain-Derived Neurotrophic Factor in the Brain of the Small-Spotted Catshark *Scyliorhinus canicula,* and Evolution of Neurotrophins in Basal Vertebrates

**DOI:** 10.3390/ijms24119495

**Published:** 2023-05-30

**Authors:** Elena Chiavacci, Sara Bagnoli, Alessandro Cellerino, Eva Terzibasi Tozzini

**Affiliations:** 1 Biology Laboratory (BIO@SNS), Scuola Normale Superiore, 56126 Pisa, Italy; elena.chiavacci@sns.it (E.C.); sara.bagnoli@sns.it (S.B.); alessandro.cellerino@sns.it (A.C.); 2 Biology and Evolution of Marine Organisms Department (BEOM), Stazione Zoologica Anton Dohrn, 80121 Napoli, Italy; 3 Fritz Lipmann Institute for Age Research, Leibniz Institute, 07745 Jena, Germany

**Keywords:** brain-derived neurotrophic factor, BDNF, neurotrophins, sea lamprey, dogfish, catshark

## Abstract

Neurotrophins (NTFs) are structurally related neurotrophic factors essential for differentiation, survival, neurite outgrowth, and the plasticity of neurons. Abnormalities associated with neurotrophin-signaling (NTF-signaling) were associated with neuropathies, neurodegenerative disorders, and age-associated cognitive decline. Among the neurotrophins, brain-derived neurotrophic factor (BDNF) has the highest expression and is expressed in mammals by specific cells throughout the brain, with particularly high expression in the hippocampus and cerebral cortex. Whole genome sequencing efforts showed that NTF signaling evolved before the evolution of Vertebrates; thus, the shared ancestor of Protostomes, Cyclostomes, and Deuterostomes must have possessed a single ortholog of neurotrophins. After the first round of whole genome duplication that occurred in the last common ancestor of Vertebrates, the presence of two neurotrophins in Agnatha was hypothesized, while the monophyletic group of cartilaginous fishes, or Chondrichthyans, was situated immediately after the second whole genome duplication round that occurred in the last common ancestor of Gnathostomes. Chondrichthyans represent the outgroup of all other living jawed vertebrates (Gnathostomes) and the sister group of Osteichthyans (comprehensive of Actinopterygians and Sarcopterygians). We were able to first identify the second neurotrophin in Agnatha. Secondly, we expanded our analysis to include the Chondrichthyans, with their strategic phylogenetic position as the most basal extant Gnathostome taxon. Results from the phylogenetic analysis confirmed the presence of four neurotrophins in the Chondrichthyans, namely the orthologs of the four mammalian neurotrophins BDNF, NGF, NT-3, and NT-4. We then proceeded to study the expression of BDNF in the adult brain of the Chondrichthyan *Scyliorhinus canicula*. Our results showed that BDNF is highly expressed in the *S. canicula* brain and that its expression is highest in the Telencephalon, while the Mesencephalic and Diencephalic areas showed expression of BDNF in isolated and well-defined cell groups. NGF was expressed at much lower levels that could be detected by PCR but not by in situ hybridization. Our results warrant further investigations in Chondrichthyans to characterize the putative ancestral function of neurotrophins in Vertebrates.

## 1. Introduction

The broad family of neurotrophic factors is comprehensive and includes neurotrophins, the GDNF family of ligands, and ciliary neurotrophic factor (CNTF) [1]. In particular, neurotrophins are a family of four closely related neurotrophic factors that comprise nerve growth factor (NGF), brain-derived neurotrophic factor (BDNF), neurotrophin-3 (NT-3), and neurotrophin-4 (NT-4) [2,3]. Neurotrophins are produced as a precursor that undergoes proteolytic processing and is subsequently released in the extracellular space [4,5,6,7]. Neurotrophins are produced by specific neuronal and non-neuronal cells in the nervous system and peripheral organs, and their synthesis and release are finely regulated during development and in adult life. Typically, neurotrophins are released by a given cell and, upon binding by specific receptors, are internalized by another cell. In the peripheral nervous system, the canonical paradigm envisions that neurotrophins are produced by non-neuronal cells in the peripheral organs and are transported by retrograde axonal transport to the cell bodies of sympathetic neurons or dorsal root ganglia neurons during a critical developmental period, determining the number of neurons that will survive programmed cell death to ensure a match between the size of the target and its innervation [8]. In the central nervous system, a key characteristic of neurotrophins is that their transcription and release are activity-dependent, and, in turn, neurotrophins regulate synaptic function and plasticity. They may also act in a paracrine/autocrine fashion or be transported anterogradely. Neurotrophins are implicated in the regulation of many aspects of neuronal function during development and in adult life, such as, for example, pain perception, food intake, circadian rhythmicity, learning and memory, and many others.

Neurotrophin signaling is mediated by specific tyrosine kinase transmembrane receptors named TrkA (for NGF), TrkB (for BDNF and NT-4), and TrkC (for NT-3), and a low-affinity neurotrophin receptor (P75 NGFR) that may act as a co-receptor or may signal autonomously.

The distribution of neurotrophins and their receptors in the central nervous system is differentiated both spatially and temporally. BDNF has the highest expression and is expressed by specific cells throughout the brain, with particularly high expression in the hippocampus and cerebral cortex [9]. NT-3 is also expressed in the hippocampus and, to a lesser extent, in the cerebral cortex, but its expression is highest in the cerebellum [10]. Notably, NT-3 expression has its peak during prenatal life, while BDNF expression is low during prenatal life and has a peak in adulthood [11,12,13]. NGF is expressed in the hippocampus, but at lower levels than BDNF or NT-3, and in the cortex. In general, contrary to BDNF and NT-3, it has been difficult to localize NGF expression by in situ hybridization in regions other than the hippocampus [14]. NT-4 is the least conserved and least expressed neurotrophin in the brain, and its expression is almost undetectable. Accordingly, mice lacking NT-4 suffer from very mild phenotypes, while knock-out of any other neurotrophin results in postnatal lethality. On note, the NT-4 gene was lost in the last ancestors of birds (https://www.ensembl.org/Homo_sapiens/Gene/SpeciesTree?db=core;g=ENSG00000225950;r=19:49061066-49065076) accessed on 16 November 2022. Consistent with the different expression of neurotrophins, TrkB and TrkC receptors have widespread, almost ubiquitous expression in the brain, whereas the expression of TrkA and p75 NGFR is limited to very specific cell types such as cholinergic neurons in the basal forebrain.

*BDNF* gene structure is characterized by the presence of multiple mutually exclusive 5′UTRs that correspond to the activation of different promoters. In humans, 10 different 5′ exons can be spliced into a common coding 3′ exon, giving rise to 34 transcripts, all coding for the same protein. Moreover, the *BDNF* locus can be transcribed bidirectionally, giving rise to antisense transcripts [15,16]. Some of these promoters are strictly activity-dependent [17], and the corresponding 5′ UTR exons control differential dendritic localization of *BDNF* transcripts [18]. Two alternative 3′ UTRs exist for *BDNF,* and these also control dendritic localization and local translation of *BDNF* [19].

From an evolutionary perspective, neurotrophins are exemplary of the two rounds of whole genome duplication (WGD) that characterized the evolution of Gnathostomes: one in the last common ancestor of Vertebrates and one in the last common ancestor of Gnathostomes. Accordingly, the genomes of Bilateria, such as, for example, the mollusk *Aplysia californica* [20] or the annelid *Platynereis dumerillii* [21], contain one neurotrophin gene, as is also the case for the Cephalochordata *Branchiostoma floridae* [22]. The two rounds of WGD occurring during the evolution of tetrapods generated the complete set of four neurotrophins, and, accordingly, we expect Chondricthyans to be the most basal vertebrate taxon possessing the complete set of four neurotrophins. Therefore, investigating their expression in the brain may reveal the basal Gnathostome condition. A third event of WGD occurred in teleosts, as reflected by the presence of a fifth neurotrophin in teleosts that is a paralog of NGF. Even if BDNF was investigated in the Actinopterygii [23,24,25], with several studies carried out in the Teleosts *Danio rerio* [26,27,28,29,30,31] and *Nothobranchius furzeri* [32,33], the divergence of the Actinopterygii and Sarcopterygii lineages from their last common ancestor occurred ~476 million years ago, according to molecular clock estimates [34]. The evolutionary ancient divergence and the presence of a further duplicated neurotrophin [35,36,37,38] declassify the ray-finned fish as not ideal whenever the origin of neurotrophins is under investigation. The introduction of model systems positioned before the occurrence of the third WGD, such as Agnatha and Chodroichtyans, is thus needed to better understand the evolution of neurotrophins in Tetrapods, potentially unveiling new strategies for the treatment of neurotrophin-related diseases.

The Chondrichthyan brain presents the conserved regions of the vertebrate brain with a large telencephalon and a well-developed cerebellum. On note, the cephalic index of Chondrichthiyans is larger than in teleosts, resulting in a cephalic index more similar to that of birds and mammals, while the allometric scaling between the different brain divisions is identical to that of primates [39,40], making neurobiological investigations in this class of particular interest. The morphogenesis of the Chondrichtyan brain is characterized by the same evagination of telencephalic vesicles that characterizes the brain morphogenesis in Tetrapods, and therefore the Chondrichthyan brain possesses two lateral ventricles.

The small-spotted catshark *Scyliorhinus canicula* is a small (<1 m) Galeomorph shark that is common in the European North Atlantic and the Mediterranean Sea. Due to its ease of collection and the possibility of captive breeding, this species arouses interest and becomes the prototype of the Chondrichthyan model. Its developmental stages are characterized and described [41], and protocols are developed for the husbandry, microinjection, and chemical treatment of the eggs [42,43,44,45,46]. More recently, a method for genetic manipulation has become available [47]. Over the years, *S. canicula* proved to be a valid and good model for the study of a lot of biological processes, such as brain development [48,49,50], lifelong tooth cycling [44,51], retina function and development [52,53,54], and the immune system [55,56,57,58]. We therefore decided to investigate the distribution of neurotrophin genes by in situ hybridization in the brain of the small spotted catshark.

To date, for the anatomical description of the *S. canicula* brain, two models are available: the classical one and the prosomeric one. Although the prosomeric model of neuroanatomical development in *S. canicula* has been proposed and is supported by some previous studies [48], we will refer to the nomenclature and anatomical distribution of the classical rostro-caudal model [59] since we focus our analysis on adult specimens, for which it is undoubtedly the most widely used reference model.

## 2. Results

### 2.1. BDNF in S. canicula and the Evolution of Neurotrophins in Cartilaginous Fishes

**Figure 1:**
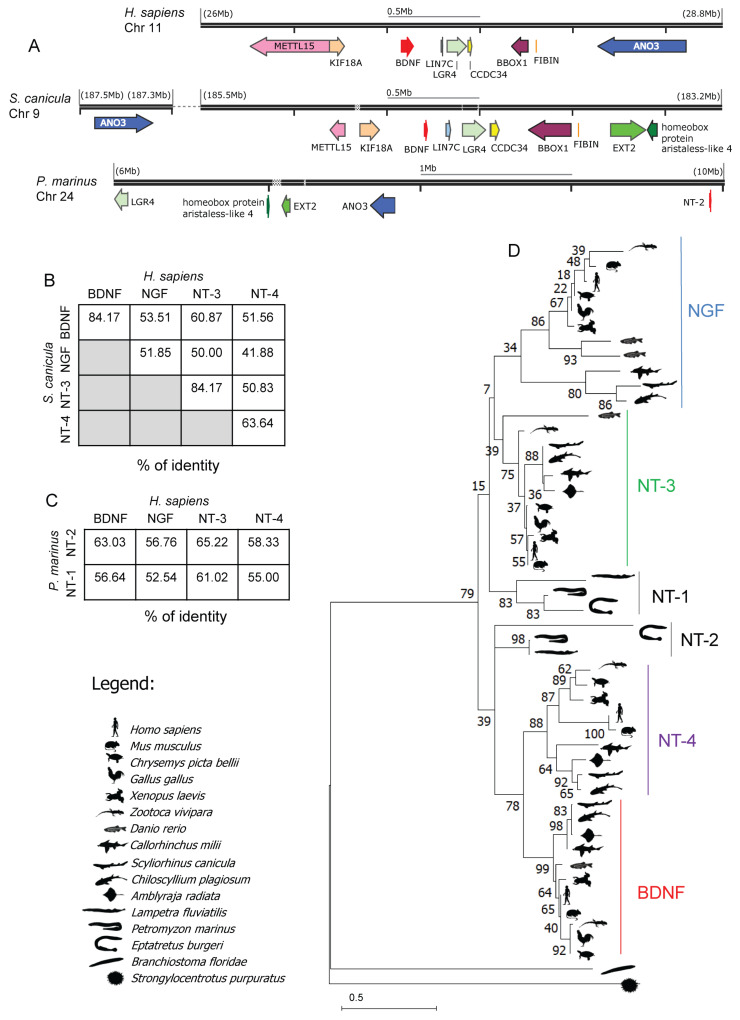
BDNF during evolution. BDNF synteny in *H. sapiens*, *S. canicula,* and *P. marinus*. Syntenic genes are color-coded (**A**). Percentage of identity between the mature peptidic sequence of the four neurotrophins in Chondrichthyan *S. canicula* and mammalian *H. sapiens* (**B**). Percentage of identity between the mature peptidic sequence of the two neurotrophins in Agnatha *P. marinus* and the four neurotrophins in mammalian *H. sapiens* (**C**). Phylogenetic tree of neurotrophin mature peptidic sequences with the invertebrate *S purpuratus* as the tree’s outgroup; species are listed on the legend (**D**).

We searched the *S. canicula* genome for *BDNF*, *NGF, NT-3,* and *NT-4*. We identified four genes, as expected. The *S. canicula BDNF* gene retains the typical structure described in mammals, presenting ten predicted mRNA splicing isoforms that differ in the 5′ UTR and all harbor the same CDS in a single exon. The analysis of the *BDNF* synteny between the Chondrichthyan *S. canicula* and the mammalian *Homo sapiens* showed an impressive degree of conservation, with eight genes positioned and oriented in the same direction in the two species and the *ANO3* gene retained in both (Figure 1A), even if flipped. Expanding the synteny analysis to the Agnatha *Petromyzon marinus,* we were able to identify a syntenic region on chromosome 24 spanning ~4 Mb upstream of one of the two *P marinus* neurotrophins that we named *NT-2*. Specifically, we found four genes conserved in *S. canicula* and two in *H. sapiens* (Figure 1A). The synteny retained around the P. marinus *NT-2* gene is the first indication suggesting this specific neurotrophin as a candidate ancestor for *BDNF*. We then proceeded to analyze the conservation of several neurotrophins.

*S. canicula* BDNF protein is highly conserved, with its mature peptidic sequence showing 84.17% identity to the mature peptidic sequence of human BDNF. The same degree of conservation was observed between *S. canicula* and human NT-3. The mature *S. canicula* NT-4 is 63.64% identical to the human NT-4, while the peptidic sequence of the mature NGF is the least conserved, with a 51.85% identity with human NGF (Figure 1B). We then investigated neurotrophin evolution among Vertebrates by reconstructing their phylogenetic tree. We collected all the peptidic neurotrophin sequences from species belonging to the Chondrichtyians class present in the NCBI and ENSEMBL genome browsers. We then discarded the partial and low-quality sequences, ending up with a total of three additional BDNF and NT-4 species-specific complete peptidic sequences in the Holocephalon *Callorhinchus milii, the* Batoid *Amblyraja radiata,* and the Galeomorph *Chiloscyllium plagiosum*, respectively. NGF was not identified in *A. radiata,* but we cannot exclude that this is due to incomplete genome annotation/assembly. For the phylogenetic analysis, we used several Gnathostomata classes as anchors, spanning from the Actinopterygii (*Danio rerio*) to the Amphibians (*Xenopus laevis*), Reptilia (*Zootoca vivipara*, *Chrysemys picta bellii*), Aves (*Gallus gallus*), and Mammalia (*Homo sapiens*, *Mus musculus*). We deliberately chose for each class the species with the largest coverage of genomic sequences in order to minimize sequencing errors and/or gaps that could affect the analysis (Supplementary Table S1).

We focused our analysis on the mature peptidic sequence, as this is more conserved and therefore better suited to resolving deep branches. As outgroups, two basal Deuteorstomata (the sea urchin *Strongylocentrotus purpuratus* and the Cephalochordata *Branchiostoma floridae*) were selected, each harboring a single neurotrophin gene. We then searched for neurotrophin genes in Agnatha and identified two genes each in the lampreys *P. marinus*, *Lampetra fluviatilis,* and hagfish *Eptatretus burgeri*. Although these Agnathan neurotrophins are correctly annotated in the respective genomes, only one of them is described in *L. fluviatilis* [60], and this is, to our knowledge, the first mention of a second neurotrophin in Agnatha.

**Figure 2:**
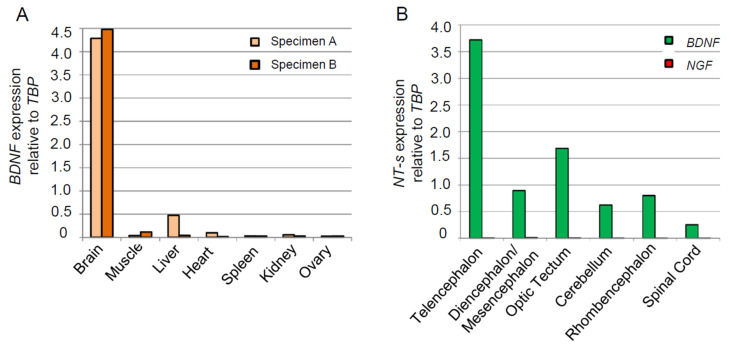
BDNF expression in *S. canicula*. BDNF expression in seven tissues of *S. canicula*, specifically the brain, muscle, liver, heart, spleen, kidney, and ovary, is relative to the housekeeping TBP (**A**). BDNF and NGF expression in the different areas of the *S. canicula* brain, relative to the housekeeping TBP (**B**).

As expected, the phylogenetic tree of Vertebrates shows a first duplication in Agnatha, with two paralog genes and an ortholog in each of the three species. The neurotrophin described by Hallböök et al. [60] in *L. fluviatilis* appears to be the common ancestor of NGF and NT-3. The mature sequence of its ortholog in *P. marinus* shows 56.64% identity with NGF (Figure 1C) and is characterized by an insertion in position 200; an insertion is also present in NT-6 of platyfish (*F. Poeciliidae*), as already described by Hallböök. This neurotrophin, which we named NT-1, is quite variable in Agnatha, also due to the presence of variable insertions. The second neurotrophin appears to be the ancestor of BDNF and NT-4, as already hypothesized by the synteny analysis. It shows a sequence identity of 63.3% with human mature BDNF (Figure 1C), and its mature sequence is identical in both Cyclostomes. For what concerns the Chondrichthyans neurotrophins, they clearly separate into four branches, each of them basal to the corresponding neurotrophin in Osteichthyes, thereby demonstrating that Chondrichthyans possess the complete set of four neurotrophins, reinforcing their value as the most basal class of vertebrates for the study of neurotrophins (Figure 1D). We then assessed *BDNF* expression in *S. canicula* by qRT-PCR. For such analysis, we extracted and measured the *BDNF* RNA levels in seven different tissues of two *S. canicula* specimens (brain, muscle, liver, heart spleen, kidney, and ovary), detecting it in all the samples, with the brain as the major expressing tissue, as expected (Figure 2A).

We decided to go further with the analysis, and we dissected a whole brain in five areas of interest, coinciding with the most common morphologically recognized brain regions (Telencephalon, Diencephalon + Mesencephalon, Optic Tectum, Cerebellum, and Rhombencephalon), plus the spinal cord. We then assessed *BDNF* and *NGF* levels in the dissected areas of the *S. canicula* brain. The results showed a preponderant *BDNF* expression in the Telencephalic area, while the second most *BDNF*-expressing region is the Optic Tectum, in agreement with *BDNF* expression in mammals. The *NGF* neurotrophin, although detectable (Telencephalon = 0.0080-fold, Diencephalon/Mesencephalon = 0.0144-fold, Optic Tectum = 0.0080-fold, Cerebellum = 0.0028-fold, Rhombencephalon = 0.0022-fold, Spinal Cord = 0.0012-fold relative to *TBP*), is expressed at a very low level in the adult *S. canicula* brain (Figure 2B).

### 2.2. BDNF Expression in the Telencephalon

We performed free-floating ISH (ff-ISH) to localize BDNF expression in the adult brain of *S. canicula*. We analyzed the entire rostro-caudal extent of the brain in two animals, one male and one female, with both showing the same staining pattern. Taking advantage of the peculiar BDNF and NGF genomic organization, we amplified the template from the genomic DNA, which is abundant and relatively simple to obtain as compared to RNA extraction and cDNA generation. We did not use sense probes as negative controls since BDNF and NGF are transcribed bidirectionally [15,16,61] and antisense transcripts were detected for these genes in mammals. Thanks to the free-floating technique adopted for the in situ, we were able to process 100-micrometer-thick sections, obtaining a signal intensity that is comparable to whole mount samples while still retaining the single-cell resolution typical of in situ performed on sections or monolayer cell cultures. We counterstained nuclei with Hoechst to mark single cells and to detect possible technical artifacts due to the unspecific binding of the probe to regions of high cellular density. As a specificity control for BDNF and NGF ff-ISH, we decided to clone and hybridize PCNA, a gene that is well known as a marker of dividing cells in neurogenic niches in Vertebrates, including fish. For the ff-ISH of NGF, we were not able to detect a signal in any of the sections we hybridized; this is not surprisingly, as NGF showed a very low expression level as compared to BDNF (Figure 2B), most likely falling under the threshold for detection of ff-ISH.

**Figure 3:**
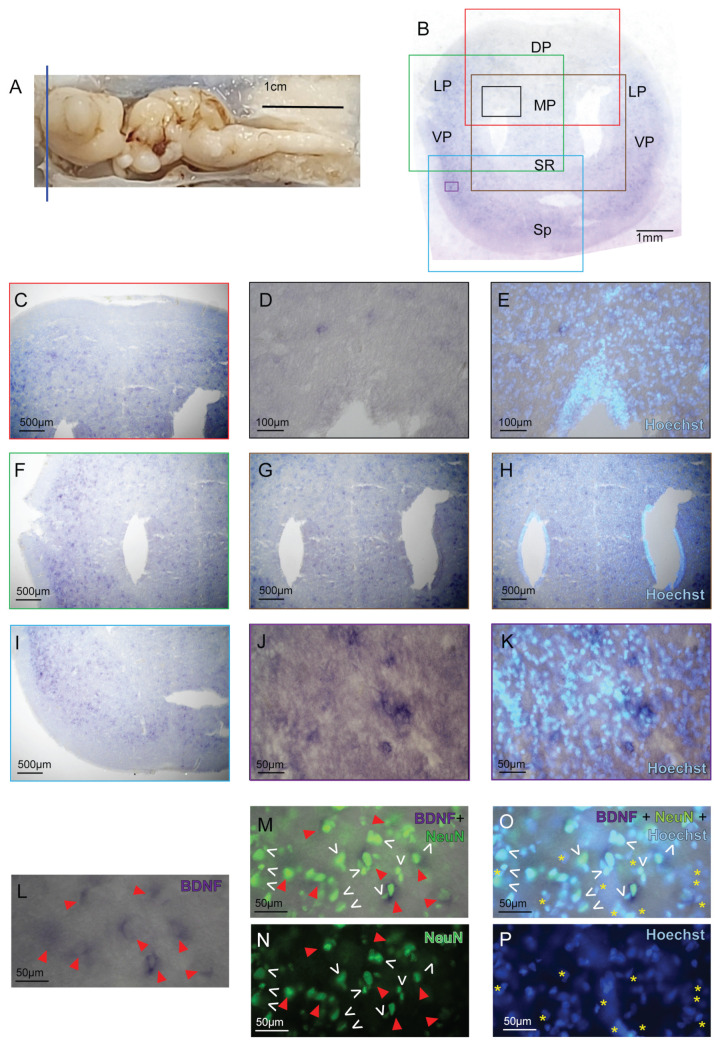
**BDNF expression in the rostral telencephalon**. *S. canicula* brain; the blue line indicates the site of the coronal sections for BDNF ff-ISH (**A**). Overview of the rostral telencephalon hybridized for BDNF (**B**). BDNF is expressed in the DP, LP (**C**–**E**), VP, MP, SR (**F**–**H**), and Sp (**I**–**K**). BDNF-positive cells (**L**–**O**, red arrows) colocalize with the neuronal nuclear marker NeuN (**M**, red arrows). For comparison, some neurons that are NeuN-positive and BDNF-negative are indicated by white arrows (**M**–**O**). Non-neuronal cells are marked by the nuclear dye Hoechst staining alone (**O**,**P** yellow asterisks). Magnifications of single areas are indicated by color codes (**B**–**K**). For abbreviations, see the list.

**Figure 4:**
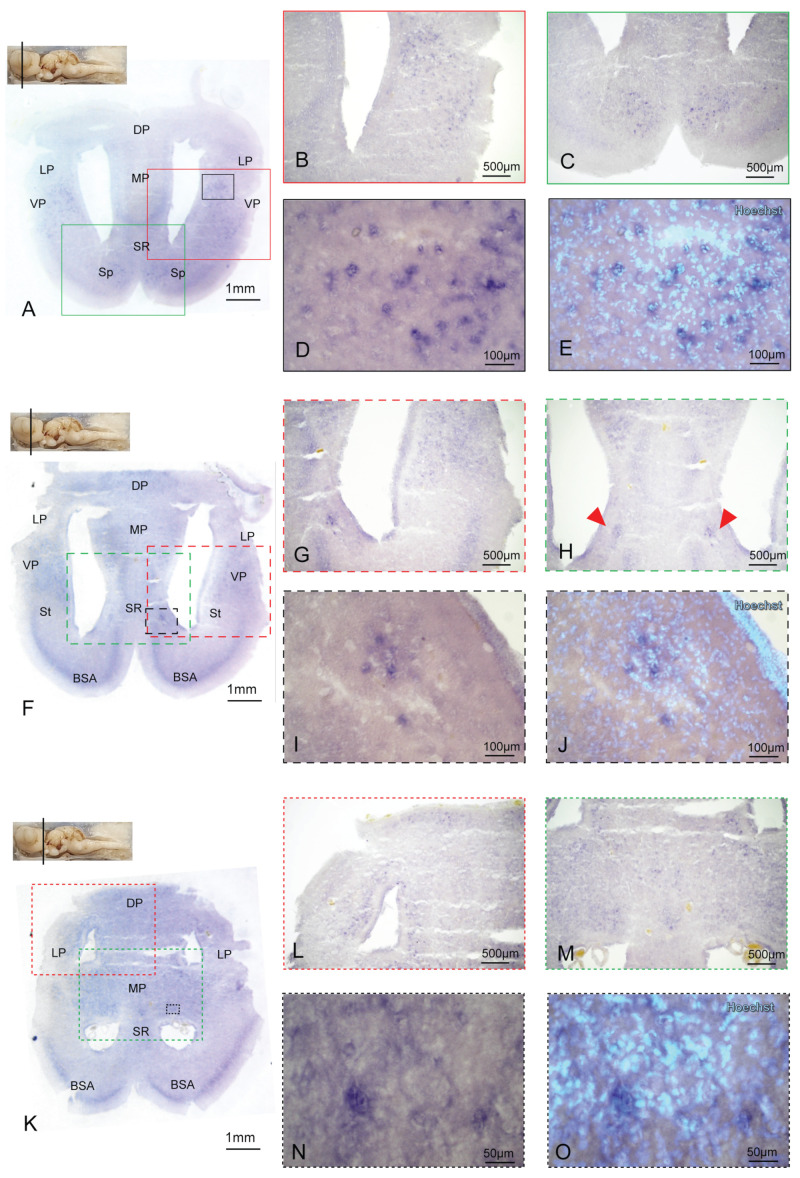
BDNF expression in the medial and posterior telencephalon. Overview of the *S. canicula* medial telencephalon hybridized for BDNF (**A**). BDNF is expressed in the VP (**B**) and Sp (**C**). Magnification at the single cell level of BDNF expression in the VP is shown in (**D**) and counterstained with Hoechst in (**E**). Overview of the medial telencephalon in correspondence with the olfactory bulbs hybridized for BDNF (**F**). BDNF expression is evident in the VP (**G**) and in a group of well-defined cells situated bilaterally in the SR region (**H**, red arrows); magnification in (**I**) is counterstained with the nuclear dye Hoechst in (**J**). Overview of the posterior telencephalon hybridized for BDNF (**K**). BDNF is expressed in the DP and MP (**L**,**M**). magnification in (**N**) is counterstained with the nuclear dye Hoechst in (**O**). Magnifications of single areas are related to color and texture codes. For abbreviations, see the list.

Still, the fact that BDNF, NGF, and PCNA riboprobes showed different hybridization patterns is a strong indication of specificity. In the most rostral portion of the telencephalon, rostral to the olfactory bulbs (Figure 3A), hybridized coronal sections of the *S. canicula* brain (Figure 3B) show extensive expression of BDNF in clearly defined cells (Figure 3B–K). This labeling is clearly not due to cell density, as BDNF-positive cells are interspersed among negative cells identified by the Hoechst nuclear counterstaining. The BDNF-positive cells are particularly abundant in the Lateral Pallium (LP), Ventral Pallium (VP), and Subpallium (Sp) (Figure 3C,F,I–K), while they are absent in the ventricular surfaces (Figure 3G,H). On the other hand, the ventricular walls are the only telencephalic regions showing scattered PCNA-positive cells (Figure S1A,B), in agreement with Bagnoli et al. [62]. In the region of the telencephalon that is midway along the rostrocaudal axis, the BDNF expression regionalizes and is restricted to specific telencephalic areas, specifically the VP (Figure 4A,B,D–G) and Sp (Figure 4A,C). To ensure the neuronal nature of the BDNF-labeled cells, we performed immunohistochemistry (IHC) on the ff-ISH with the well-known and established postmitotic neuronal nuclear marker NeuN [63,64]. Results showed that all the cells expressing BDNF are positive for NeuN (Figure 3L–P). In the same fashion, we detected some other neurons that are not BDNF-positive (Figure 3M,O, white arrows). Non-neuronal cells labeled by Hoechst only (Figure 3O,P, yellow asterisks) were present as well. These results undoubtedly revealed the nature of the BDNF-positive cells, identifying them as a subpopulation of mature neurons.

**Figure 5:**
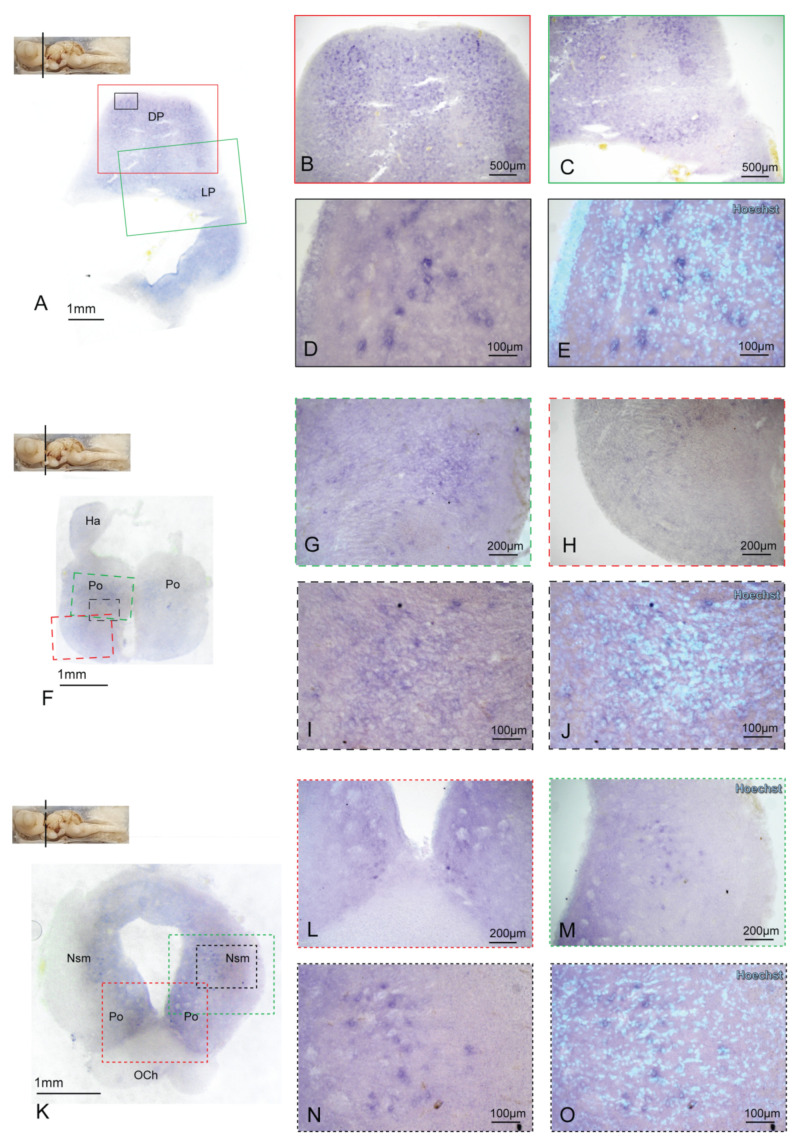
BDNF expression in the posterior Telencephalon and mesencephalic Po. Overview of the *S. canicula* most caudal portion of the telencephalic region hybridized for BDNF (**A**). BDNF expression is evident in the DP and LP (**B**,**C**). Magnification in (**D**) is counterstained with the nuclear dye Hoechst in (**E**). Overview of the mesencephalic Po hybridized for BDNF (**F**,**K**). BDNF-positive cells are present in the Po areas (**G**–**J**,**L**,**N**,**O**) and the Nsm (**M**). Magnifications are counterstained with the nuclear dye Hoechst in (**J**,**O**). Magnifications of single areas are related to color and texture codes. For abbreviations, see the list.

In agreement with what is already known in mice [65,66], BDNF is expressed at very low levels in the Striatum (St), resulting in no detectable signal (Figure 4F,G), while a group of few well-defined cells bilaterally positioned in the Septal Region (SR) was revealed to be BDNF-positive (Figure 4H–J). The DP and Medial Pallium (MP) also start to show BDNF expression in this coronal section, and the expression is maintained until the most caudal telencephalic area (Figure 4F,K–O and Figure 5A–E).

The “V”-shaped area crossing the DP, MP, and SR, resembling a possible signal, proved to be a retention of background due to the high cellular density in such a region (Figure S2B–E). We detected the same unspecific labeling of cells in the thick cell band of the basal superficial area (BSA) (Figure 4F,K and Figure S3A,D,E) and inside the Entopeduncular nucleus (En) (Figure S4A–C).

### 2.3. BDNF Expression in the Diencephalon and Mesencephalon

**Figure 6:**
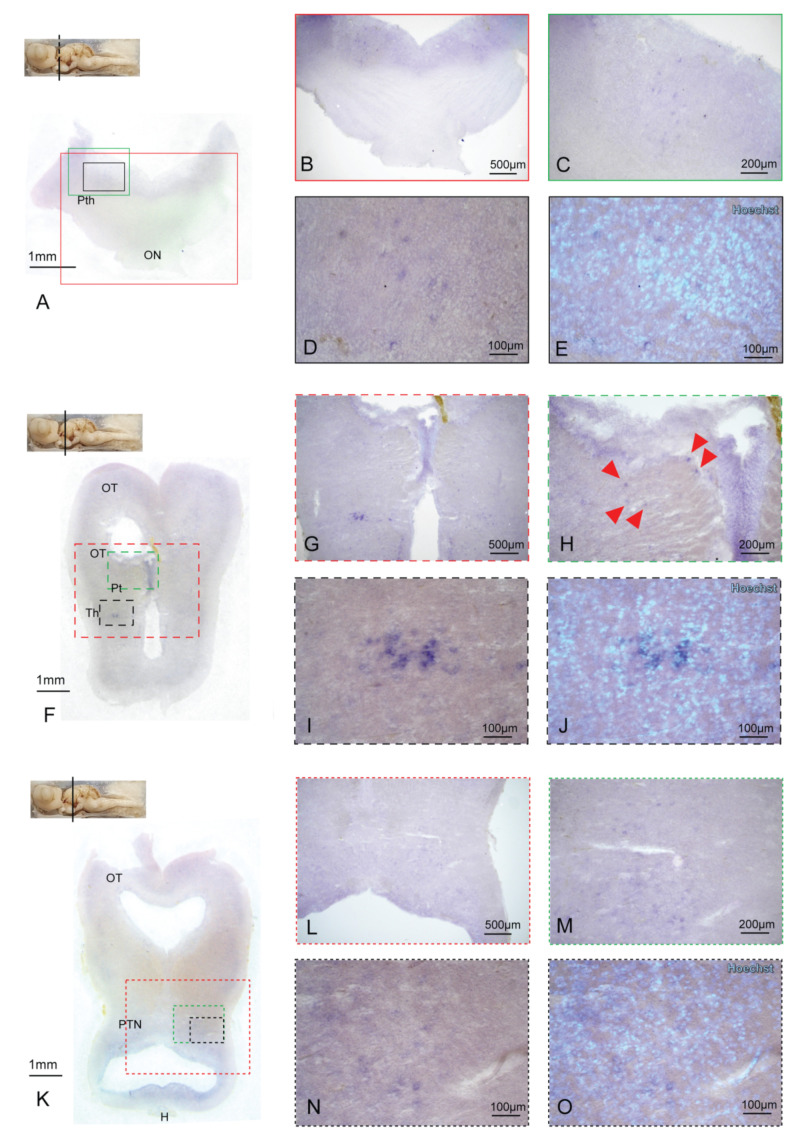
BDNF expression in the Diencephalon and Mesencephalon. Overview of the *S. canicula* diencephalic and mesencephalic areas hybridized for BDNF (**A**,**F**,**K**). BDNF-positive cells were detected in the Pth (**B**–**E**), Pt, and OT (**H**, red arrows), as well as in the Th areas (**G**). Magnification in (**I**) is counterstained with the nuclear dye Hoechst in (**J**). BDNF expression was detected in the PTN areas too (**L**–**O**), with magnification in (**N**) counterstained with the nuclear dye Hoechst in (**O**). Magnifications of single areas are related to color and texture codes. For abbreviations, see the list.

The mesencephalic Preoptic area (Po) presents some BDNF-expressing cells homogeneously scattered in the structure (Figure 5F–J). BDNF expression narrowed, moving towards a more caudal section. At the beginning of Optic Chiasm (OCh), BDNF can be detected in the Po ventral region (Figure 5K,J) and in the Nuclei of the stria medullaris (Nsm) (Figure 5K,M–O). Some BDNF-positive cells are then distributed along the diencephalic Prethalamus (Pth) (Figure 6A–E) and the mesencephalic Thalamus (Th) (Figure 6F,G,I,J).

**Figure 7:**
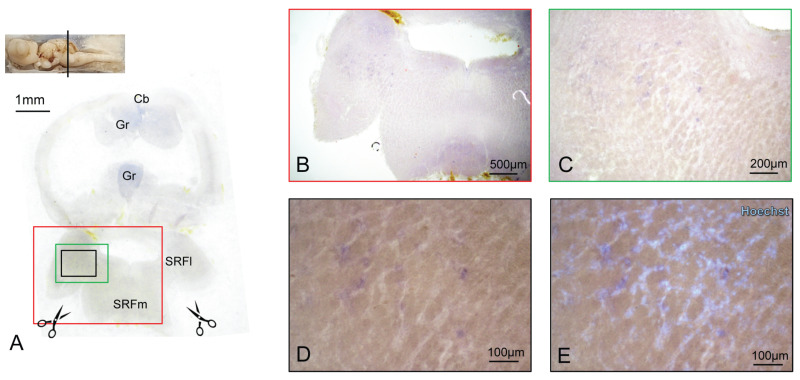
BDNF expression in the SRFI and SRFm. Overview of the *S. canicula* SRFI and SRFm hybridized for BDNF. To obtain a better overview of the hybridized tissue, two cuts were made in correspondence with the scissors logo (**A**). BDNF-positive cells were detected in the SRFI and SRFm areas (**B**,**C**); magnification in (**D**) was counterstained with the nuclear dye Hoechst in (**E**).

BDNF-expressing cells are detectable in the Pretectum too (Pt) (Figure 6H). The Posterior Tubercle Nuclei (PTN) are a further mesencephalic BDNF-expressing area, with cells scattered throughout the entire structure (Figure 6K–O). Some BDNF-expressing cells are detectable in the OT (Figure 6H), in agreement with what was already observed in other models [67]. The most caudal region of *S. canicula* brain in which we were able to detect BDNF-positive cells via ff-ISH was the lateral part of the superior reticular formation (SRFl) (Figure 7A–E), while no BDNF expression was detected in the Cerebellum (Cb) (Figure 7A and S5B–E).

The cerebellar neurogenic niches retain background staining due to their extreme cellular density, which can be appreciated via Hoechst counterstaining (Figure S5B–D). We also failed to detect expression of BDNF in the Rhombencephalic areas (Figure S5A–G).

## 3. Discussion

In the present paper, we present the first analysis of BDNF expression in the Galeaomoph shark, *S. canicular,* as a representative of Chondrichthyan. Chondrichthyans are the most basal group of Vertebrates, and here we show that they possess the complete set of four neurotrophins, so the analysis of their expression is of particular interest.

Our phylogenetic analysis of the neurotrophin coding sequence reveals that events of whole genome duplication that occurred during the evolution of vertebrates are faithfully represented in the neurotrophin evolutionary tree. The previous version of the *P. marinus* genome (Petromyzon_marinus-7.0, GenBank accession number #GCA_000148955.1) was released in 2010, and the quality of the sequencing was at scaffold level (https://www.ncbi.nlm.nih.gov/datasets/genome/?taxon=7757 accessed on 27 April 2023), leaving a certain degree of uncertainty in genetic and phylogenetic studies. In 2020, thanks to the Vertebrate Genomes Project, a new version of the *P. marinus* genome was released (kPetMar1, GenBank accession number #GCA_010993595.1). The kPetMar1 was assembled at chromosome level, and its size is 1,1 Gb, compared to the 885.5 Mb of the Petromyzon_marinus-7.0. The release of the new genome allows us to perform a consistent and reliable phylogenetic analysis of the origin of neurotrophins. The presence in the sequenced genome of *P. marinus* of two neurotrophins is of particular interest, and it marks a key milestone in the evolution of neurotrophic signaling, possibly associated with an initial differentiation in their functions. The second event of neurotrophin gene duplication, likely associated with their final functional diversification, took place in the last common ancestor of all Gnathostomes, and, accordingly, Chondrichthyans possess four neurotrophins in their genomes, a condition maintained in all Tetrapods with the exception of a secondary loss of NT-4 in the bird lineage. All these occurrences are beautifully recapitulated in the phylogenetic tree shown in Figure 1D. Moreover, the analysis of the synteny around the *H. sapiens* and *S. canicula* BDNF loci denoted impressive conservation. Although the linear order of a genome segment containing a set of genes may have been shuffled considerably during evolution, the extremely high synteny level around the *H. sapiens* and *S. canicula* BDNF genes allows us to identify a further syntenic region in the Agnatha *P. marinus*, specifically around the neurotrophin NT-2 gene locus. Our comprehensive data, including the BDNF locus syntenic analysis and the neurotrophins phylogenetic tree reconstruction, confirmed that the BDNF locus present in Tetrapods originated from a common ancestor through the 1st and 2nd rounds of duplication events that occurred during the evolution of Vertebrates. The pattern of conservation of neurotrophin sequences in basal Vertebrates follows what was already described [35]. BDNF and NT-3 are highly conserved, while NGF and NT-4 are more variable in their sequence.

Our analysis reveals a deep level of conservation concerning BDNF expression. Firstly, *S. canicula* BDNF is more expressed in the brain as opposed to peripheral organs and is the most expressed neurotrophin in the brain. Second, *S. canicula* BDNF shows several different 5′ exons corresponding to different 5′ UTRs, and it is highly likely that these correspond to transcription from different promoters, as well described in humans and rodents [16,68].

We then performed in situ hybridization to localize expression of *S. canicula* BDNF at the cellular level. Since BDNF is transcribed bidirectionally, the option of using a sense probe as a control is suboptimal. We therefore compared the expression of *S. canicula* BDNF with that of *S. canicula* PCNA, which is specifically expressed in the neurogenic niches of the brain. On the other hand, a probe for *S. canicula* NGF did not result in any clear cellular staining. The comparison between these three signals shows that they label different cell types with no overlap. In addition, the labeling for *S. canicula* BDNF is limited to defined cells with clear staining surrounded by cells devoid of staining. For this reason, we assume that our in situ methodology specifically identifies cells expressing *S. canicula* BDNF. In the description of the staining, we concentrated on the cells with high expression; obviously, we cannot exclude that other cell types express *S. canicula* BDNF at lower levels, undetectable by our methodology. Indeed, we failed to detect cells expressing *S. canicula* NGF even though this gene is expressed, albeit at low levels, in the *S. canicula* brain, as shown by qRT-PCR.

*S. canicula* BDNF expression is particularly high in the Telencephalon and, in particular, in regions of the Pallium. In mammals, the regions with the highest expression of BDNF are the cortices and the hippocampus. BDNF is also expressed in the Telencephalon of songbirds and the dorsal pallium of zebrafish and killifish. Therefore, telencephalic expression of BDNF appears to be a conserved basal trait of Gnathostomes. Further, in mammals, it is well characterized that BDNF is not expressed in the basal ganglia, where it is transported anterogradely by the cortical cells [66]. Similarly, we do not observe expression of *S. canicula* BDNF in the telencephalic region that is supposed to represent the homolog of Striatum. It is of note that we detected an expression of BDNF in the Optic Tectum, in accordance with the fact that retinal ganglion cells are known to depend on the expression of BDNF in their target in the Optic Tectum. Although BDNF expression was detected in the Cb and in the rhombencephalic areas via qRT-PCR, we were not able to detect any BDNF-positive cells via ff-ISH in such areas, indicating that the BDNF expression levels in those areas are most likely due to basal expression, falling below the ff-ISH detection threshold.

In summary, our study lays the foundation and the motivation to investigate neurotrophin function in the CNS of Chondrichtyans to characterize their putative ancestral functions.

## 4. Materials and Methods

### 4.1. Synteny and Phylogenetic Analysis

Synteny analysis was carried out by identifying ortholog genes via Blastp alignments of peptidic sequences, and visualization of results was obtained with Snapgene software ver. 6.2.1. Inference of NT-s evolutionary relationships was performed using NT-s peptidic sequences of different species obtained from NCBI (https://www.ncbi.nlm.nih.gov/ accessed on 1 December 2021) and Ensembl genome browser 106 (https://www.ensembl.org/index.html accessed on 1 December 2021). Entries are listed in Supplementary Table S1. Mature peptidic sequences were aligned with Basic Local Alignment Search Tool ver. Blastp (https://blast.ncbi.nlm.nih.gov/Blast.cgi?PAGE=Proteins accessed on 1 December 2021). The phylogenetic tree was constructed with MEGA11 Molecular Evolutionary Genetics Analysis version 11 (https://www.megasoftware.net/, accessed on 1 December 2021) as follows: sequences of mature NT-s were aligned with ClustalW, and the maximum likelihood method was applied to the JTT + G model. The model was chosen as the best based on the Akaike Information Criteria. The cladograms and robustness were estimated at each branching node by 100 random bootstrap replications. The phylogenetic view of NT’s evolutionary relationships was obtained by MEGA11 as an output.

### 4.2. Tissue Processing

Adult specimens of *S. canicula* were supplied alive by local fishermen; the animals were sacrificed, and the brains were immediately dissected and fixed overnight in PFA 4% in compliance with and approved by the Italian Ministry of Health (Cod. B290E.N.TU2). For the free floating in situ hybridization (ff-ISH): following decapitation, the brains were dissected from the skull, postfixed overnight (ON) in paraformaldehyde (PFA) at 4 °C in PBS, rinsed twice in PBS, then equilibrated in sucrose 25% until the tissue sank to the tube bottom (a minimum of 6 h). The tissues were then embedded in Tissue-Tek O.C.T. Compound (Sakura, Torrance, CA, USA) and 100-micrometer-thick sections were cut on microscope glass slides by Menzel-Glaser (Thermo Fisher Scientific, Waltham, MA, USA). Slides with sections were stored at −20 °C until hybridization. For RNA extraction, the fresh dissected brain, muscle, and liver were sunk in QIAzol (Qiagen, Venlo, The Netherlands) immediately after dissection and stored at −80 °C until extraction. For genomic DNA extraction, muscle samples were freshly dissected and frozen at −20 °C until extraction.

### 4.3. Total RNA Extraction and RT-qPCR

Total RNA from several tissues from two different specimens was extracted using the RNeasy Mini kit (Qiagen) according to the manufacturer’s protocol and quantified using a FC-3100 (NanoReady, Shaanxi, China) spectrophotometer; quality was checked by electrophoresis on an agarose gel in RNAse-free conditions. One µg of each RNA was retrotranscribed for cDNA synthesis with the QuantiTect Reverse Transcription Kit (Qiagen). Such kits have a DNAse treatment step specific to avoiding genomic contamination. qPCR was performed with SsoAdvanced™ Universal SYBR^®^ Green Supermix (Biorad Hercules, CA, USA) and 35ng of cDNA for each sample as a template in Rotorgene equipped with Rotorgene Q software ver 2.1.0.9 (Qiagen). Relative gene quantification was calculated using the ΔΔCt method with *TBP* as the reference gene. Primer sequences are *TBP* Fw: 5′-AGACAATAGCCCTTCGAGCA-3′, *TBP* Re: 5′-TTCTTGCAGCCAATCGTGAC-3′, *BDNF* Fw: 5′-GTGAGCGTCCTGGAGAAGAT-3′, *BDNF* Re: 5′-TATCCATAGTTAGGGCGCGC-3′, *NGF* Fw: 5′-GCCTCAAGCGGAATGACAAT-3′, *NGF* Re: 5′-TCGAACCAGTGTCCCATTCA-3′

### 4.4. Genomic DNA Extraction and DIG-Labeled Riboprobe Synthesis

For DIG-labeled probe synthesis, genomic DNA extraction was performed with the DNeasy Blood and Tissue Kit (Qiagen) according to the manufacturer’s protocol, and *BDNF* template was PCR amplified from genomic DNA using a forward Fw: 5′-CCAATGGAAGAAGCAGGAGG-3′ and a reverse primer carrying a T7 promoter sequence, here underlined, on its 5′ end, Re: 5′-TAATACGACTCACTATAGGGCCTTATAAATCTCCACCCAA-3′. *NGF* template was PCR amplified from genomic DNA using a forward Fw: 5′-ATGTCCATCTTTTACTCGTT-3′ and a reverse primer carrying a T7 promoter sequence on its 5′ end, Re: 5′-TAATACGACTCACTATAGGGTCACGGTTTCCATGTTTTCC-3′. For *PCNA* riboprobe, the template was obtained by PCR amplification from cDNA using a forward Fw: 5′-GCTCTACCGGCATCAGTTTG-3′ and a reverse primer carrying a T7 promoter sequence, here underlined, on its 5′ end, Re: 5′-TAATACGACTCACTATAGGGTGAAGAAGTTCAGGTACC-3′. The PCR product was purified with Wizard^®^ SV Gel and PCR Clean-Up System (Promega), verified via Sanger Sequencing (Eurofins Genomics), and then 50 ng were used as a template to be directly transcribed with T7 RNA polymerase (Thermofisher Scientific) and digoxygenated RNTP mix (Roche) for 2 h at 37 °C. The resulting DIG-labeled riboprobes were precipitated with 1/10 volume of LiCl (5 M) and 2.5 volumes of ethanol ON at −20 °C, washed with 75% ethanol, resuspended in nuclease-free water, and stored at −80 °C.

### 4.5. Free Floating In Situ Hybridization (ff-ISH) and Immunohistochemistry (IHC)

ff-ISH was performed according to Thisse et al. [69] with some modifications. Sections were rehydrated in PBS, detached from the glass slice, and recovered in 2 mL safelock Eppendorf tubes, one section each. Sections were directly pre-hybridized for 30 min at 66 °C and then incubated with a digoxigenin DIG-labeled probe at 66 °C ON. Immediately before incubation, the probe was denatured at 80 °C for 3 min. Sections were washed for 15 min at 66 °C twice, first with SSC-2x and then with SSC-0.2x. Three washes in PBST at RT followed, and then sections were incubated with anti-Dig AP Fab (Roche) 1/2000 at 4 °C ON in a blocking solution (Roche, Basel, Switzerland). The day after, sections were washed three times for 5 min in PBST at RT, then moved into a 24-well plate, one section per well. Sections were treated with TMN solution (Tris-MgCl2-NaCl buffer) three times for 5 min, then stained with BM-Purple (Roche). The staining was constantly monitored under a stereomicroscope (M80 Leica) equipped with an LED light O-ring and blocked by washing in PBST. Once the color was fully developed, sections were post-fixed in PFA 4% ON and nuclei stained with Hoechst 33342 (Invitrogen, Waltham, MA, USA) 1:5000 for 5 min. Immunohistochemistry was performed as follows: ff-ISH (not Hoechst-treated) were washed three times in PBS and then blocked with a solution containing 5% BSA + 0.3% Triton-X 100 in PBS for two hours at RT. Sections were then incubated overnight at 4 °C with NeuN primary antibody 1:500 diluted (Abcam #Ab177487). Sections were rinsed three times in PBS and incubated with a secondary antibody 1:500 diluted for 2 h at RT (Invitrogen #A11001). Sections were then rinsed again three times in PBS 1, and nuclei were counterstained with Hoechst 1:5000 in PBS at RT for 5 min. Sections for ff-ISH or ff-ISH + IHC were then mounted on Superfrost Plus glass slides (Thermo Fisher Scientific) with fluoroshield mounting medium and coverslipped. Whole panoramic view images were acquired with a stereomicroscope (M80 Leica) equipped with an Axiocam ERc 5S (Zeiss, Oberkochen, Germany) and an LED light O-ring. Magnification and fluorescence images were acquired using an epifluorescence microscope (Nikon Eclipse600) equipped with a DS-Fi3 color camera (Nikon, Tokyo, Japan) and a double LED light O-ring. Images were processed with Gimp 2.10.32 and ImageJ software.

## Supplementary Materials

The following supporting information can be downloaded at: https://www.mdpi.com/article/10.3390/ijms24119495/s1.

## Abbreviations

BSA	Basal Superficial Area
Cb	Cerebellum
DON	Dorsal Octavolateralis Nucleus
DP	Dorsal Pallium
En	Entopeduncular nucleus
Gr	Granular zone
H	hypophysis
Ist	Isthmus
IRF	Inferior Reticular Formation
LP	Lateral Pallium
LX	Vagal Lobe
MP	Medial Pallium
MRF	Middle Reticular Formations
Nsm	Nucleus of the stria medullaris
OCh	Optic Chiasm
ON	Optic nerve
OT	Optic Tectum
Pth	Prethalamus
PTN	Posterior Tubercle Nucleus
Po	Preoptic area
Sp	Subpallium
SR	Septal Region
SRFl	Superior Reticular Formation, lateral
SRFm	Superior Reticular Formation, medial
St	Striatum
Tg	Tegmentum
Th	Thalamus
VP	Ventral Pallium
