# Supplementary material for: Distribution of Brain-Derived Neurotrophic Factor in the Brain of the Small-Spotted Catshark Scyliorhinus canicula, and Evolution of Neurotrophins in Basal Vertebrates
Source: Int J Mol Sci. 2023 May 30;24(11):9495. doi: 10.3390/ijms24119495 (PMC10253963; doi:10.3390/ijms24119495)
Supplement: Supplementary file 1 [file ijms-24-09495-s001.zip › ijms-2373915-supplementary.pdf]

Table S1. List of the ID entries used for the phylogenetic analysis.

Species	Entry ID
Strongylocentrotus purpuratus	NP_001073024.1
Branchiostoma floridae	XP_035663096.1
Petromyzon marinus	XP_032815947.1
Petromyzon marinus	XP_032833900.1
Lampetra fluviatilis	CBX24522.1
Lampetra fluviatilis	AAD22744.1
Eptatretus burgeri	ENSEBUT00000021255.1
Eptatretus burgeri	ENSEBUT00000018613.1
Homo sapiens	NP_001700.2
Homo sapiens	NP_002497.2
Homo sapiens	NP_001096124.1
Homo sapiens	NP_006170.1
Xenopus laevis	XP_041445004.1
Xenopus laevis	XP_018104654.1
Xenopus laevis	XP_018106340.1
Xenopus laevis	XP_041427551.1
Gallus gallus	XP_015141634.1
Gallus gallus	NP_001280037.1
Gallus gallus	NP_001103232.1
Zootoca vivipara	XP_034973699.1
Zootoca vivipara	XP_034979280.1
Zootoca vivipara	XP_034993816.1
Zootoca vivipara	XP_034992115.1
Mus musculus	NP_031566.4
Mus musculus	NP_001106168.1
Mus musculus	NP_001157506.1
Mus musculus	NP_937833.1
Chrysemys picta bellii	XP_005306427.1
Chrysemys picta bellii	XP_005294994.1
Chrysemys picta bellii	XP_005296635.1

Chrysemys picta bellii	XP_005315481.2
Danio rerio	NP_001295577.1
Danio rerio	NP_001314742.1
Danio rerio	NP_001338647.1
Danio rerio	NP_571139.1
Scyliorhinus canicula	XP_038676845.1
Scyliorhinus canicula	XP_038639559.1
Scyliorhinus canicula	XP_038667129.1
Scyliorhinus canicula	XP_038664236.1
Callorhinchus milii	XP_007885859.1
Callorhinchus milii	XP_007902351.1
Callorhinchus milii	NP_001280089.1
Callorhinchus milii	NP_001280081.1
Chiloscyllium plagiosum	XP_043561376.1
Chiloscyllium plagiosum	XP_043569511.1
Chiloscyllium plagiosum	XP_043573031.1
Chiloscyllium plagiosum	XP_043535539.1
Amblyraja radiata	XP_032894526.1
Amblyraja radiata	XP_032895486.1
Amblyraja radiata	XP_032869089.1
