# Supplementary material for: Distribution of Brain-Derived Neurotrophic Factor in the Brain of the Small-Spotted Catshark Scyliorhinus canicula, and Evolution of Neurotrophins in Basal Vertebrates
Source: Int J Mol Sci. 2023 May 30;24(11):9495. doi: 10.3390/ijms24119495 (PMC10253963; doi:10.3390/ijms24119495)
Supplement: Supplementary file 1 [file ijms-24-09495-s001.zip › BDNF_paper_Supplementary_Sub.pdf]

Supplementary data

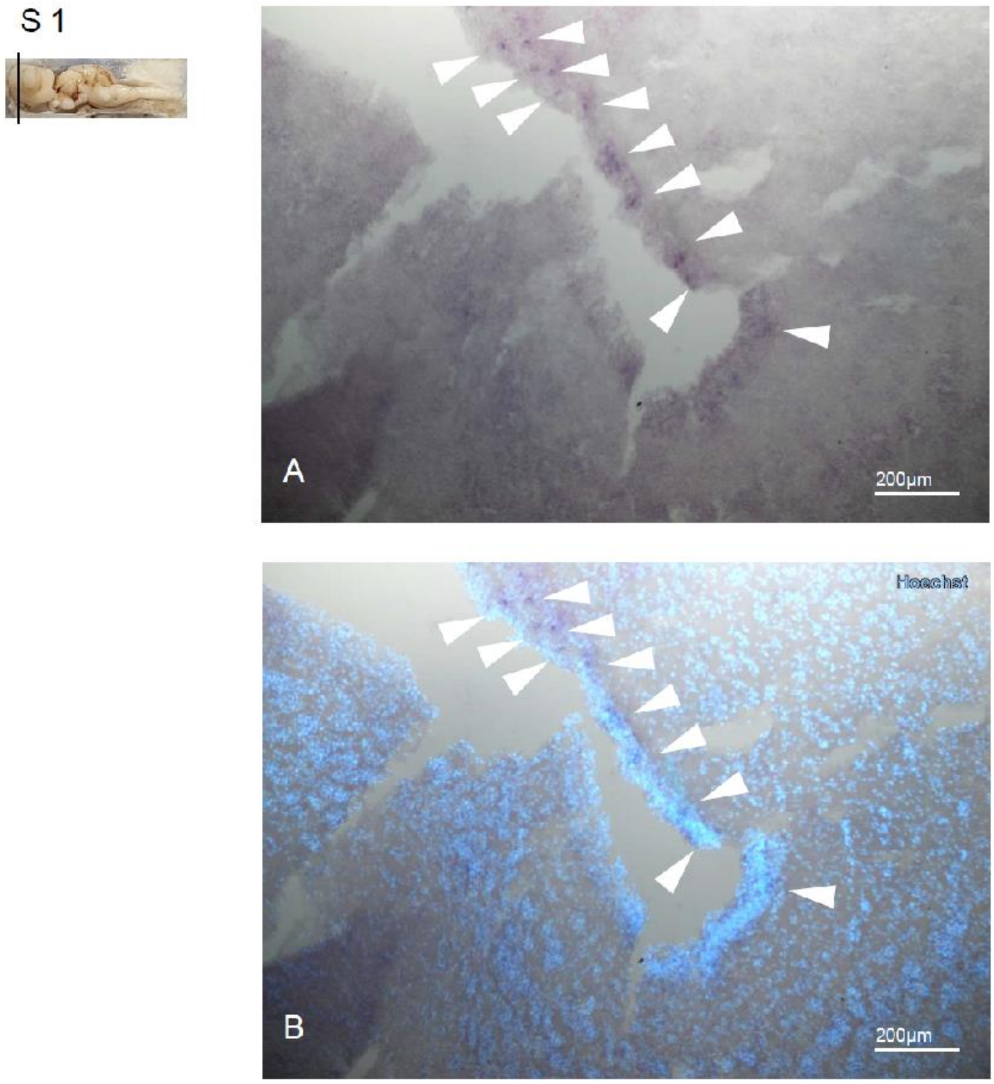

Figure S1. PCNA expression in the telencephalon of *S. canicula*, the black line indicate the site of the coronal sections for PCNA ff-ISH, PCNA-positive cells are presents in the ventricular walls **(A, B** white arrows**)**, nuclei are counterstained with Hoechst **(B)**.

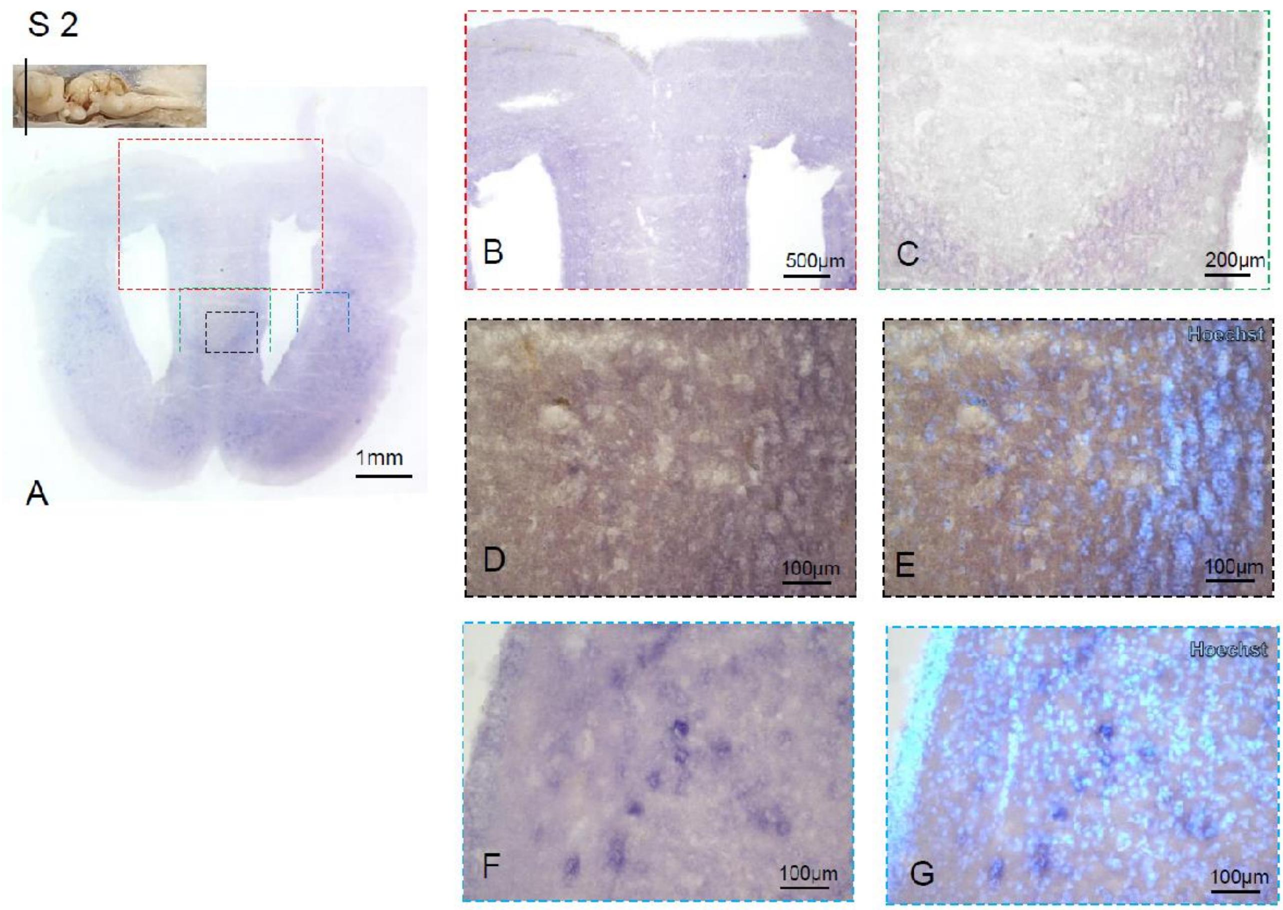

Figure S2. *S. canicula* telencephalic region, the unspecific background labelling is visible in the cell-dense “V” shaped area crossing the DP, MP and SR **(A-E)**, while the BDNF-positive cells are clearly distinguishable in VP **(F-G)**. Nuclei are counterstained with Hoechst **(E;G)**. Magnifications of single areas are respective of color- and texture- code. For abbreviations see list.

S 3

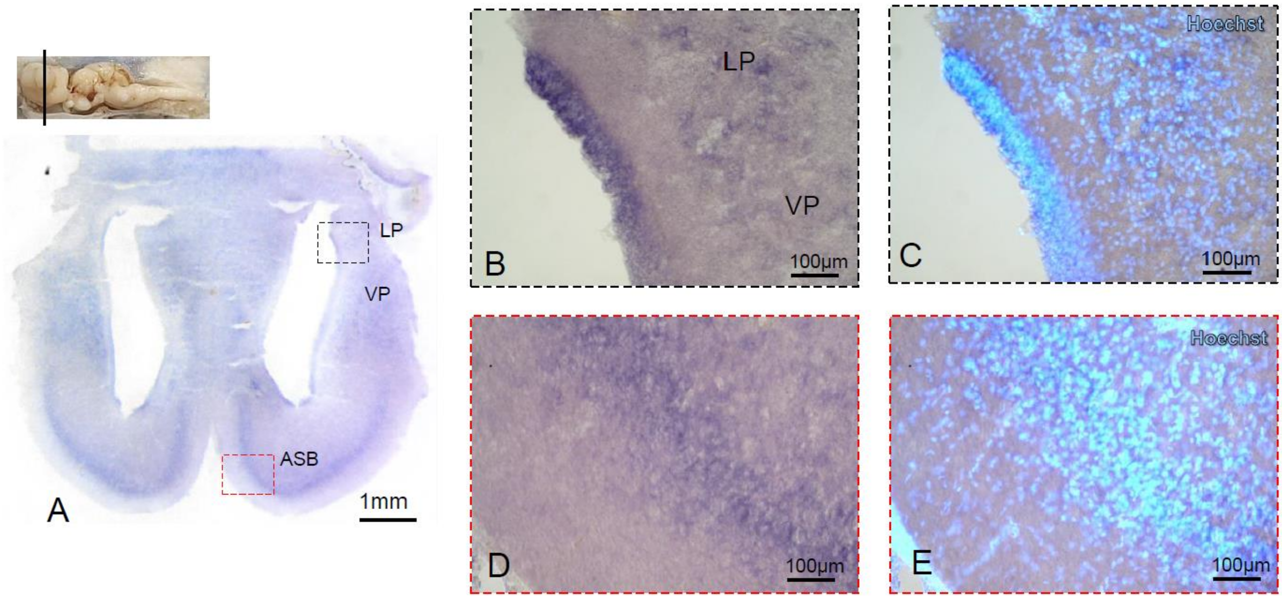

Figure S3. *S. canicula* medial telencephalic region, the unspecific background labelling is visible in the cell-dense ASB area **(A; D; E)** while the BDNF-positive cells are clearly distinguishable in LP and VP **(A-C)**. Nuclei are counterstained with Hoechst **(C;E)**. Magnifications of single areas are respective of color- and texture- code. For abbreviations see list.

S 4

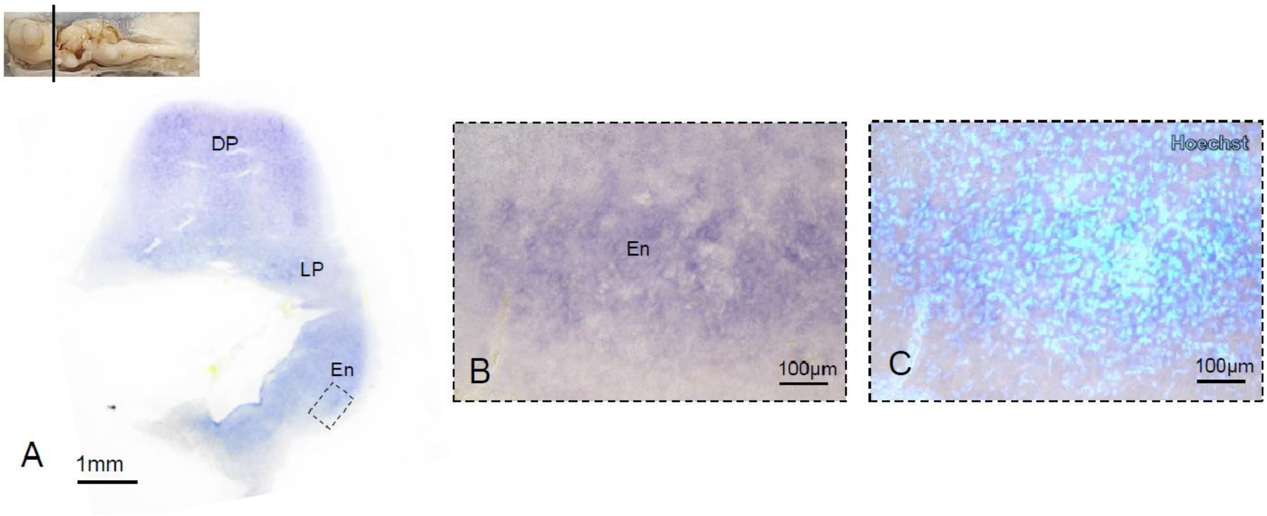

Figure S4. *S. canicula* posterior telencephalic region, the unspecific background labelling is visible in the cell-dense En area **(A-C)**. Nuclei are counterstained with Hoechst **(C)**. Magnifications of single areas are respective of color- and texture- code. For abbreviations see list.

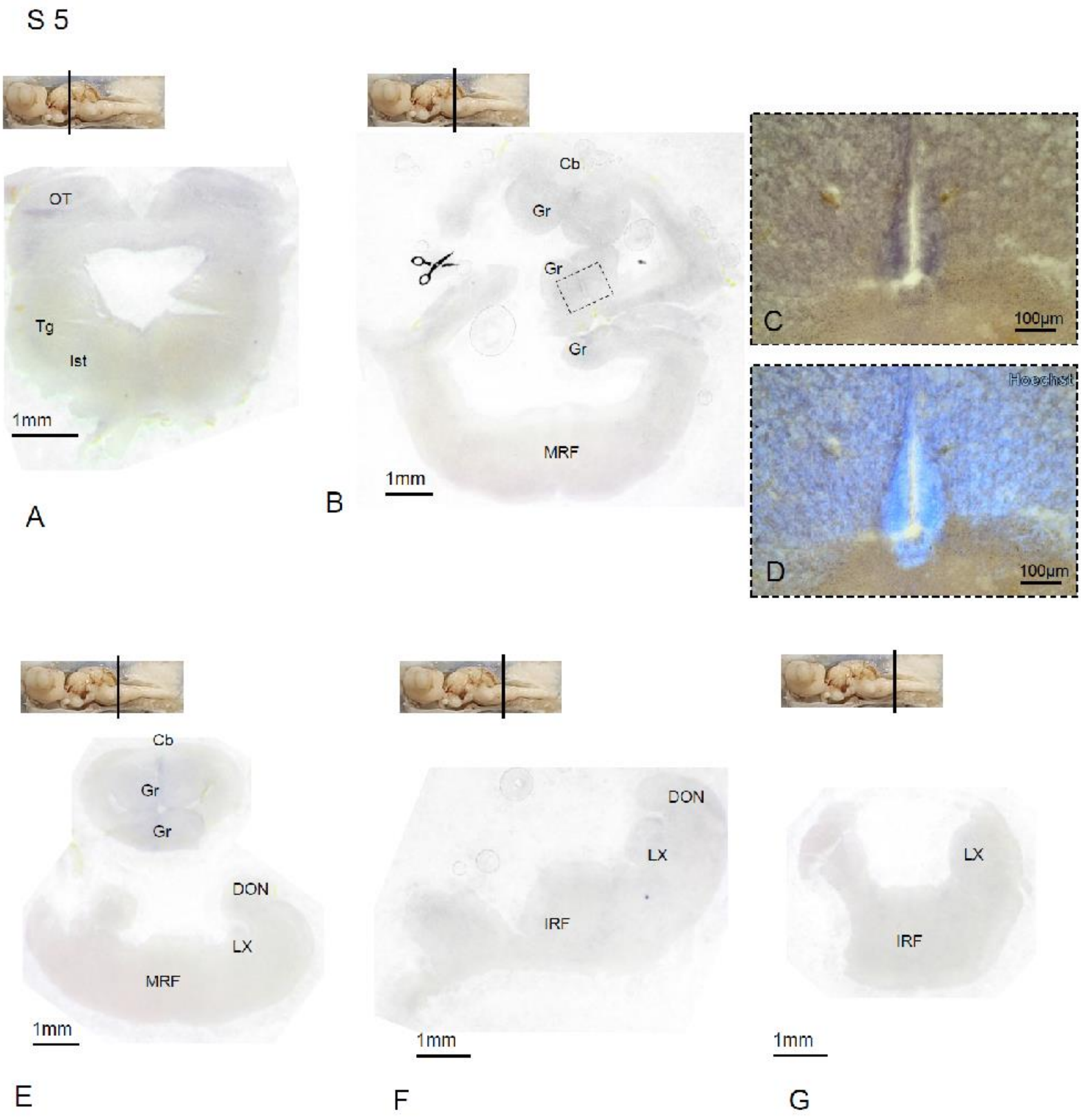

Figure S5. *S. canicula* rhombencephalic areas **(A, B)**. The cerebellar neurogenic niches retain background due to their extreme cellular density **(C, D)**, as highlighted by Hoechst nuclear counterstain **(D)**. No BDNF-positive cells were detected in the rhombencephalon **(E-G)**. To obtain a better overview of the hybridized tissue a cut was made in correspondence with the scissor logo. Magnifications of single areas are respective of color- and texture- code. For abbreviations see list.
